# Autoantibodies in Myositis. How to Achieve a Comprehensive Strategy for Serological Testing

**DOI:** 10.31138/mjr.30.3.155

**Published:** 2019-09-30

**Authors:** Miriam Mende, Viola Borchardt-Lohölter, Wolfgang Meyer, Thomas Scheper, Wolfgang Schlumberger

**Affiliations:** EUROIMMUN AG, Luebeck, Germany

**Keywords:** autoantibodies, autoimmunity, dermatomyositis, inclusion body myositis, myositis, polymyositis, serology

## Abstract

Myopathies are a rare type of acquired, chronic autoimmune diseases of the skeletal muscles and affect both children and adults. The hallmark symptoms of idiopathic inflammatory myopathies (IIM) are muscle inflammation, proximal muscle weakness and disability, arthritis, cutaneous rashes, calcinosis, ulceration, malignancy and interstitial lung disease (ILD). Subforms of IIM include polymyositis, dermatomyositis, cancer-related myositis and sporadic inclusion body myositis. Autoantibodies function as biomarkers for diagnosis of IIM and can be used to delimit clinically distinguishable IIM subforms. To maximise the diagnostic information it is essential to perform comprehensive multiparametric serological testing including both screening and confirmation tests.

## SUBFORMS OF IIM

Polymyositis (PM) is a systemic inflammatory disease of the skeletal musculature with perivascular lymphocytic infiltration. Clinical symptoms of PM are recurring bouts of fever, muscle weakness, arthralgia, Raynaud syndrome and trouble with swallowing. When the skin is involved, the disease is known as dermatomyositis (DM). In DM, skin symptoms appear as purple-coloured exanthema on the eyelids, nose and cheeks, periorbital oedema, local erythema and scaly eczema dermatitis.^1^ DM and PM are separate diseases with different pathophysiological mechanisms and are clinically diagnosed according to standard criteria by Bohan and Peter.^2^

Both PM and DM can be associated with a paraneoplastic syndrome.^3,4^ Large population-based studies have shown a tumour frequency of 20–25%, with a higher occurrence in DM than in PM.^5^ Adenocarcinomas are the most common tumours in myositis, representing about 70% of malignancies.^6^ Although adenocarcinomas are the most common type of cancer in DM, an increased risk is associated with all histological types.^3^ DM is strongly associated with ovarian, lung, pancreatic, stomach and colorectal cancers and non-Hodgkin lymphoma, while PM is associated with an increased risk of lung and bladder cancers and non-Hodgkin lymphoma.^3^ In the course of disease, DM symptoms usually occur before the tumour is detectable. For all cancer types, there was a three-fold higher risk of malignant disease after diagnosis of DM.^3^ Most tumours are detected within one year following myositis diagnosis, but DM patients are still at increased risk for malignancy five years later. In both subforms, risk of malignant disease is highest at time of myositis diagnosis.^3^ According to the modified Bohan and Peter classification, cancer-associated myositis was defined as cancer occurring in patients within three years of diagnosing myositis.^7^ In general, the risk to develop a malignancy increases with patient’s age.^5,8,9^ As another subform of IIM, myopathies with perimysial pathology show a combination of damage to perimysial connective tissue and muscle fibre necrosis. This pathology is associated with an increased risk of ILD, Raynaud syndrome, mechanic’s hands and inflammatory arthritis.^10^ Among the IIMs, anti-synthetase syndrome (ASS) is a severe condition characterised by extramuscular and multiple organ involvement, affecting especially the lungs. The classical triad manifestations of ASS are myositis, ILD and non-erosive arthritis.^11,12^ Patients suffering from the subform necrotising myopathy rapidly develop progressive muscle weakness and severe debilitation within months of onset.^13^ Necrotising myopathy has been observed in patients previously treated of cardiovascular disease with statins.^14^ Statin-associated autoimmune myopathy is a very rare side effect of statin use and is related to presence of autoantibodies against HMG-CoA reductase.^15^ However, in a large cohort study one third of patients with autoimmune myopathy, who have never been prescribed statin therapy, tested positive for anti-HMG-CoA reductase autoantibodies.^16^

Sporadic inclusion body myositis (sIBM) is a rare subform of IIM and difficult to distinguish from other subforms.^17^ It is a degenerative autoimmune disease of the muscles accompanied with inflammatory infiltrates and inclusion vacuoles. Its prevalence varies between different populations and amounts to 1 to 71 per million individuals, rising to 139 per million in people older than 50 years.^18^ Clinical manifestations of sIBM are dysphagia, muscle weakness and atrophy, preferentially affecting the quadriceps femoris and the wrist and finger flexors.^19^ The disease is chronic and slowly progressive, leading to severe disability.^9^ First-line therapy for DM, PM and sIBM are corticosteroids, but second-line immunosuppressive agents (prednisone, azathioprine, methotrexate, mycophenolate) are often required in addition.^20^

## AUTOANTIBODIES IN IIM AND THEIR DIAGNOSTIC RELEVANCE

IIM cases that are suspected on clinical grounds are currently confirmed by muscle biopsy, magnetic resonance imaging or electromyography.^21^ Disadvantages of obtaining muscle biopsies are that the extraction necessitates anaesthesia, and may lead to adverse effects. Alternatively, IIM can be characterised based on analysis of blood samples. A major advantage of blood-based testing compared to muscle biopsies is its comparatively low degree of invasiveness as well as the broad range of additional testing possibilities based on one collected sample. Evidence of general blood-based laboratory testing in IIM encompass increased muscle enzyme values and unspecific signs of inflammation, such as increased C-reactive protein titer and acceleration of erythrocyte sedimentation rate. Based on these advantages, it is recommended to always initially perform serological testing followed by investigation of a biopsy sample if necessary.

Diverse autoantibodies are associated with myositis, targeting nuclear and cytoplasmic components of the cell and increasing evidence suggests that patients with PM and DM have specific clinico-serological profiles (*Table 1*).^22^ The autoantibody specificity correlates with pathogenesis and indicates distinct clinical manifestations.^23^ The autoantibodies are divided into myositis-specific autoantibodies (MSA), which are found primarily in patients with IIM, and myositis-associated autoantibodies (MAA), which are unspecific for the disease but are nevertheless important diagnostic markers.^24^ Newer classification strategies are now utilising MSA as biomarkers, which correlate with clinical and histopathological phenotypes and risk of malignancy, and help in offering prognostic information with regard to treatment response.^20^ Target antigens of MSA include the nuclear antigens Mi-2α, Mi-2β, SAE1, NXP2, MDA5, cN-1A and TIF1γ and the cytoplasmic antigens Jo-1 PL-7, PL-12, EJ, OJ, signal recognition particle (SRP), and further tRNA synthetases.^25^ Target antigens of MAA are the nuclear antigens Ku, PM-Scl75, PM-Scl100 and the cytoplasmic antigen Ro-52.

**Table 1.** Diagnostic relevance of autoantibodies in myositis.

Myositis-specific autoantibodies	Associated subform of IIM	Myositis-associated autoantibodies	Associated subform of IIM
Mi-2α	DM, caDM	Ku	OS
Mi-2β	DM, caDM	PM-Scl75	OS, DM
SAE1	DM, ILD, caDM	PM-Scl100	OS, DM
NXP2	DM, ILD, caDM	Ro-52	ILD
MDA5	DM, ILD		
cN-1A	sIBM		
TIF1γ	DM, caDM		
Jo-1	ASS		
PL-7	ASS, ILD		
PL-12	ASS, ILD		
EJ	ASS, ILD		
OJ	ASS, ILD		
SRP	ASS, necrotising myopathy, cardiac involvement		

ASS: anti-synthetase syndrome; caDM: cancer-associated dermatomyositis; DM: dermatomyositis; OS: overlap syndromes; sIBM: sporadic inclusion body myositis.

Autoantibodies against Jo-1, PL-7, PL-12, EJ, and OJ are characteristic of ASS, while those against Mi-2α, Mi-2β, SAE1, NXP2, MDA5 and TIF1γ occur in DM.^26,27^

MSAs are the most useful parameter differentiating IIM from non-IIM patients with a specificity of 95% endorsing their credentials as valuable disease biomarkers.^28,29^ However, a recent study based on a cohort of 1637 myositis patients identified that 38% had no identifiable MSA or MAA.^29^ Multiple IIM autoantibodies were detected in 55% of the patients in a longitudinal study.^30^ The most common autoantibodies are anti-Ro52 (30% of patients), anti-Ku (23%), anti-synthetases (22%), anti-U1RNP (15%), and anti-fibrillarin (14%).^34^ Rare autoantibodies like anti-EJ and anti-OJ have a low prevalence of 1%^22,25,31,32^. To achieve a successful treatment outcome, it is important to accessorily screen for rare autoantibodies. For example, anti-OJ autoantibodies can be found in patients with ILD, either in isolation or in combination with IIM. A possible existence of anti-OJ autoantibodies should be considered in patients with severe muscle involvement.^33^ Patients with the anti-EJ type of ASS demonstrated higher rates of recurrence.^34^ However, the distribution of autoantibodies is influenced by age and ethnic group. For example, anti-Jo-1 antibodies are highly prevalent in adult IIM patients and are associated with the ASS.^13,29^ Other MSA related to ASS are anti-PL-7, anti-PL-12, anti-EJ, anti-OJ, anti-KS, anti-Zo, anti-Ha-YRS, and anti-SRP.^35^ Anti-SRP autoantibodies are further associated with severe necrotising myopathy.^13^

Based on both the clinical symptoms and the detectable autoantibodies, some patients could be assigned to more than one autoimmune disease category. Such overlap syndromes between IIM and additional organ-specific autoimmune syndromes are frequently seen, for example with myasthenia gravis, autoimmune hepatitis, primary sclerosing cholangitis, Hashimoto’s thyroiditis and diabetes mellitus. For example, existence of antibodies against PM-Scl75, PM-Scl100 and Ku indicate an overlap syndrome, in particular with the autoimmune connective tissue disease systemic sclerosis. Clinically, it is useful to decode overlap syndromes to establish prognosis and facilitate treatment.

Positivity for myositis autoantibodies can also be the first indicator of an underlying tumour. Autoantibodies against anti-SAE1, anti-TIF1-γ and anti-NXP2 are associated with an increased risk of cancer in adult patients with IIM, whereas in some cases production of anti-HMGCR, anti-Jo-1 and anti-PL-12 autoantibodies may also be driven by malignancy.^36^ In consequence, it is recommended to always follow-up any myositis diagnosis in adults by tumour screening. Autoantibodies targeting the melanoma differentiation-associated protein 5 (MDA5), which appear in patients with DM and mild or absent muscle inflammation, entail an increased risk of ILD and severe vasculopathy.^37^ The MDA5 autoantibody is also a specific biomarker for early diagnosis of juvenile DM associated with ILD.^38^ The autoantibodies anti-Ro52, -MDA5, -PL7, -PL12, -OJ and -EJ, are associated with an elevated risk of ILD.^39,40^ A recent investigation of a Chinese cohort reports MSA detection rates to be 82% and 88% in 17 PM patients with ILD and 57 DM patients with ILD, respectively.^41^ Therefore, screening for ILD in case of a confirmed DM diagnosis is advisable. Despite being only a highly unspecific MAA, anti-Ro-52 is of diagnostic value as it gives meaningful additional information about disease prognosis and outcome. For example, anti-Ro-52 frequently co-occurs in anti-Jo-1-positive patients and hints towards an increased risk of mechanic hands, malignancy, and a decreased functional status at long-term follow-up.^42^

## SEROLOGIC TESTING VIA AUTOANTIBODY PROFILING

**Figure 1.**
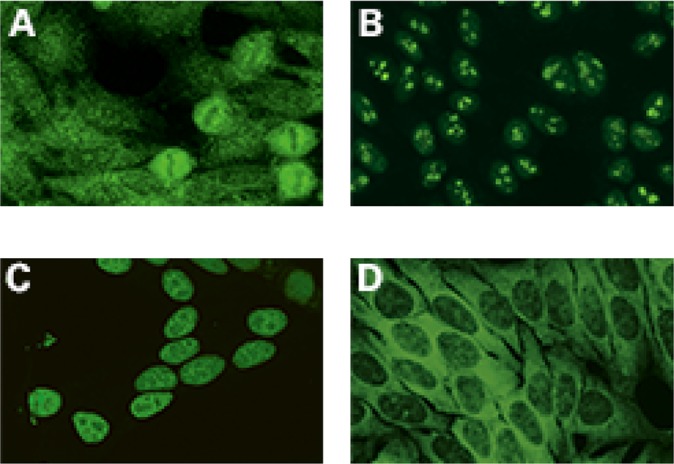
Indirect immunofluorescence screening test results on Hep-2 cells of four selected autoantibodies. (**A**) Autoantibodies against Jo-1 show a fine granular cytoplasmatic fluorescence. (**B**) Autoantibodies against PM-Scl show a homogeneous fluorescence in the nucleoli. (**C**) Anti-Ku autoantibodies show a granular pattern in the nucleoplasm. (**D**) Anti-PL-7 autoantibodies show a homogeneous fluorescence in the cytoplasm.

To serologically investigate samples of patients with suspected PM or DM, indirect immunofluorescence tests (IIFT) are used. Due to its high sensitivity and specificity, the IIFT with human epithelial cells and primate liver is the gold standard screening test for the detection of anti-nuclear autoantibodies. As an example, *Figure 1* shows IIFT results positive for four selected autoantibodies. Screening with IIFT should be followed by a confirmation of results by monospecific tests. Because antibodies against the cytoplasmic antigens are sometimes not clearly detectable with IIFT, performance of both the screening and confirmatory test is recommended. Immunoblots are an ideal confirmatory method, as they enable qualitative, monospecific but simultaneous detection of many different antibodies. Line blots allow antigens with widely differing properties to be combined on one test strip, allowing profiles to be assembled according to the disease application. A line blot contains several membrane chips printed with individual lines of antigens and the intensity of the line colour reaction correlates with the antibody titer. To achieve a high serological hit rate, rare autoantibodies with a low prevalence should be included in testing.^43^ Sequential testing has the disadvantages of being time-consuming, inefficient and potentially fragmentary. Therefore, comprehensive multiparametric testing including screening and confirmation tests is essential to maximise the diagnostic information obtained from the blood sample.

**Figure 2.**
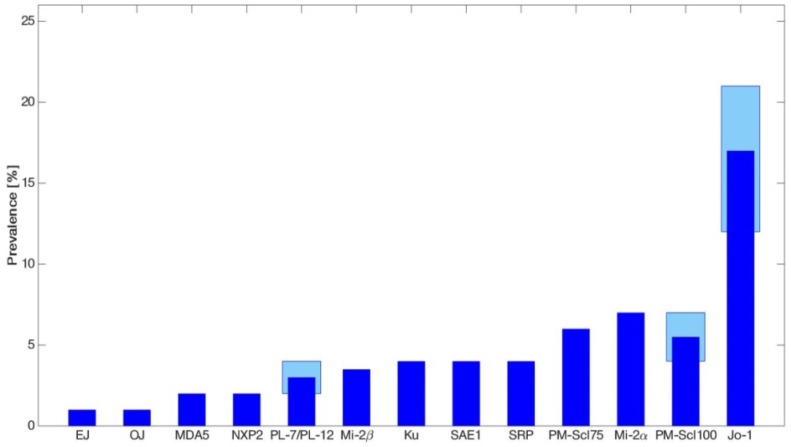
Prevalences of autoantibodies in myositis were derived from studies including in total 804 myositis patients.^22,25,31^ Light blue rectangles depict range of respective prevalence. Note that all prevalences are below 25%.

In three unrelated studies, a total of 804 sera from myositis patients and 786 control sera were investigated with a line blot containing Mi-2α, Mi-2β, TIF1γ, MDA5, NXP2, SAE1, Ku, PM-Scl100, PM-Scl75, Jo-1, SRP, PL-7, PL-12, EJ, OJ and Ro-52 on one test strip [EUROLINE Autoimmune Inflammatory Myopathies 16 Ag, EUROIMMUN, Lübeck, Germany].^22,25,31^ The specificities for the individual antigens were between 97–100%. The autoantibody prevalences were however low indicating that even the more common parameters occur only sporadically und clearly underpin the need for comprehensive multiparametric testing including screening and confirmation tests (*Figure 2*).

Comprehensive studies in various centres in Europe have indeed shown that the simultaneous investigation of various MSA in a large profile significantly increases the serological detection rate. Cavazzana et al analysed the performance of the EUROLINE Autoimmune Inflammatory Myopathies 15 Ag (IgG) [EUROIMMUN, Lübeck, Germany] for the identification of autoantibodies in sera of patients affected by myositis, compared with immunoprecipitation.^44^ The use of immunoprecipitation is restricted to few research laboratories, mainly because it uses radioisotopes, but also because it is labour and time-consuming and requires a highly specific training in interpretation of the results. Furthermore, immunoprecipitation does not differentiate between antibodies targeting proteins with the same molecular weight. In contrast, line blot assays are easy and fast to use and interpretation of results is straightforward. Using the line blot assay allowed the detection of anti-MDA5, anti-OJ and anti-TIF1γ MSA that were previously not found with routine methods.^44^ The line blot assay is valid, specific and useful to identify subgroups of IIM with specific clinical features and in accordance with the European League Against Rheumatism.^45^

## DIAGNOSTIC RELEVANCE OF ANTI-CN-1A AUTOANTIBODIES

The clinical distinction between the subforms of IIM is challenging but crucial for therapeutic decisions. SIBM is poorly responsive to conventional immunotherapies and has a high misdiagnosis rate and a mean delay to diagnosis of 5 to 8 years.^46^ The only currently known serological biomarker for sIBM are autoantibodies against the skeletal muscle antigen cytosolic 5′-nucleotidase 1A (cN-1A).^43,47^ Due to their high specificity, their detection can in particular aid the differentiation of sIBM from other muscle diseases such as PM, DM, necrotising myopathy, muscular dystrophy or myasthenia gravis.^48^ The detection of anti-cN-1A autoantibodies may facilitate the early diagnosis of sIBM, especially when the clinical picture is unclear and/or when typical pathological features are not yet detectable.^18^ Anti-cN-1A testing can play a valuable role in securing an early diagnosis and reducing the number of muscle biopsies per person.

In a recent study the diagnostic performance of the Anti-cN-1A ELISA [EUROIMMUN, Lübeck, Germany] was evaluated by two reference laboratories using different serum panels.^18^ The first cohort consisted of a total of 286 sera from patients with clinically and pathologically diagnosed definite sIBM, patients with suspected sIBM, myositis controls, non-myositis autoimmune controls and healthy subjects. The second cohort comprised a total of 253 sera from patients with definite sIBM and from healthy controls. Anti-cN-1A reactivity was most frequent in cases of definite sIBM (39% to 47%), but absent in biopsy-proven PM or DM. Overall the diagnostic sensitivity amounted to 36% in the first cohort and 39% in the second, while the specificity was 96% and 97%, respectively. Importantly, the sensitivity and specificity measured at the two different laboratories were highly similar. The study thus confirms the high specificity of anti-cN-1A antibodies for sIBM and their utility for differentiating sIBM from other IIMs. Rietveld and colleagues determined the occurrence of anti-cN-1A reactivity in patients with primary Sjörgen’s syndrome and systemic lupus erythematosus being of moderate prevalence using the Anti-cN-1A ELISA.^49^ However, as patients with sIBM and those with primary Sjörgen’s syndrome and systemic lupus erythematosus are straightforward to differentiate clinically and immunologically, this co-occurrence is of minor relevance for clinical practice.^50,51^

## SUMMARY AND OUTLOOK

Diagnosing PM or DM is challenging due to rarity of the diseases, their similar clinical presentation and the possibility of overlap syndromes. A comprehensive strategy for serological testing comprises parallel determination of both MSA and MAA to reduce the time to diagnosis as well as including as many parameters as possible to ensure the highest detection rate. Nevertheless, a negative serological test result does not exclude the presence of a disease, as exemplified by the general low prevalences of autoantibodies in IIM. For diagnosis, the clinical symptoms of the patient and further relevant evidence should always be taken into account alongside the serological test results. Importantly, due to the association of DM and PM with a variety of tumours, it is recommended to follow-up any IIM positive test result with a targeted screening for malignancies. The early identification of patients without classical myopathy features but increased risk of potentially life-threatening complications such as ILD is an aim, whereby the strong associations of MSA with specific clinical features may be of help. For multiparametric confirmatory testing, immunoblots are ideal as they offer broad antigen combinations, easy interpretation and full automatability. Further studies are in progress to probe the clinical meaningfulness of anti-cN-1A determination, for example the association of anti-cN-1A antibodies with particular disease features. It is anticipated that ongoing research will identify further novel autoantibodies related to myositis, enabling the diagnostic possibilities to be expanded further. Further studies might identify novel autoantigenic targets in myositis patients who are currently regarded as autoantibody-negative. Further work to establish the role of autoantibodies in the pathogenicity of myositis will be required. In the same vein, the exploration of specific myositis autoantibodies as predictors of disease course and treatment responses will continue in future. It is to be expected that the clinical definition of myositis subforms will undergo a development in the coming years, mirroring the progress in research. In future, specific autoantibody combinations may predict more IIM subforms than the traditional PM and DM variants.
